# Habit strength and between-meal snacking in daily life: the moderating role of level of education

**DOI:** 10.1017/S1368980018001283

**Published:** 2018-05-29

**Authors:** Saskia Wouters, Viviane Thewissen, Mira Duif, Rob JH van Bree, Lilian Lechner, Nele Jacobs

**Affiliations:** 1 Faculty of Psychology & Educational Sciences, Open University of the Netherlands, Valkenburgerweg 177, 6419 AT Heerlen, The Netherlands; 2 Department of Psychiatry and Neuropsychology, European Graduate School for Neuroscience, SEARCH, Maastricht University Medical Centre, Maastricht, The Netherlands

**Keywords:** Habit, Snacking, Experience sampling method, Ecological momentary assessment, Level of education

## Abstract

**Objective:**

Recent research emphasizes the importance of habit in explaining patterns of energy intake and choices of consumption. However, the nature of the association between habit strength and snacking has not been explored for all types of between-meal snacks.

**Design:**

Multilevel linear techniques were used to: (i) examine the association between habit strength and moment-to-moment energy intake (kilocalories) from snacks in daily life; and (ii) determine whether gender, age, level of education and BMI moderate the association between habit strength and moment-to-moment energy intake from snacks. A smartphone application based on the experience sampling method was used to map momentary between-meal snack intake in the context of daily life. Demographics and habit strength were assessed with an online composite questionnaire.

**Setting:**

This research was performed in the Netherlands in the natural environment of participants’ daily life.

**Subjects:**

Adults (*n* 269) aged 20–50 years.

**Results:**

Habit strength was significantly associated with moment-to-moment energy intake from between-meal snacks in daily life: the higher the strength of habit to snack between meals, the higher the amount of momentary energy intake from snacks. The association between habit strength and moment-to-moment energy intake from snacks was moderated by education level. Additional analyses showed that habit strength was significantly associated with moment-to-moment energy intake from between-meal snacks in the low to middle level of education group.

**Conclusions:**

It is recommended to address habitual between-meal snacking in future interventions targeting low- to middle-educated individuals.

Worldwide, the number of people with overweight and obesity has increased substantially over the last three decades and the expectation is that this trend will continue^(^ ^1^ ^)^. Despite efforts to prevent and reduce overweight and obesity, these conditions are estimated to afflict 1·9 billion (52 %) adults globally^(^ ^1^ ^)^. Research has often identified energy intake as the driving force of the rapid increase in overweight individuals^(^ ^2^ ^,^ ^3^ ^)^. Different dietary factors, such as the intake of sugar-sweetened beverages^(^ ^4^ ^)^, increased portion sizes^(^ ^5^ ^,^ ^6^ ^)^, eating away from home^(^ ^7^ ^)^ and/or consumptions with higher energy density (e.g. energy drinks), have contributed to overconsumption of energy^(^ ^8^ ^)^. The consumption of snacks has often been identified as an important contributor to the rapid increase in overweight individuals^(^ ^2^ ^,^ ^8^ ^–^ ^12^ ^)^. Nevertheless, the association between snacking and body weight has also yielded contradictory results^(^ ^11^ ^,^ ^13^ ^,^ ^14^ ^)^. For instance, a review on the association between snacking and weight in adults found inverse correlations between snacking and abdominal obesity^(^ ^11^ ^)^ although it has been suggested that this may be due to under-reporting of snack consumption^(^ ^11^ ^)^. Moreover, the term ‘snack’ is heavily debated^(^ ^13^ ^,^ ^15^ ^)^. Dietary research has employed different definitions of a snack^(^ ^13^ ^,^ ^15^ ^)^. Some research advocates the exclusion of beverages^(^ ^13^ ^)^, whereas other research postulates that in modern industrialized societies, the term ‘snack’ refers to all types of foods (including fruit) and drinks consumed outside the context of main meals (i.e. breakfast, lunch and dinner)^(^ ^9^ ^)^.

Nowadays, all types of consumptions are omnipresent and easily accessible. As a consequence, the desire to snack can be satisfied immediately^(^ ^16^ ^–^ ^18^ ^)^. The availability of increased portion sizes (e.g. king-size chocolate bars, large cups of soft drinks) and/or consumptions with higher energy density contributes to overconsumption of energy^(^ ^5^ ^,^ ^6^ ^,^ ^16^ ^,^ ^19^ ^,^ ^20^ ^)^. Recent research emphasizes the importance of habit in explaining patterns of energy intake and consumption choices^(^ ^21^ ^–^ ^23^ ^)^. Habits develop when learned sequences of acts, performed in stable contexts, have been reinforced in the past by rewarding experiences^(^ ^24^ ^–^ ^26^ ^)^. The more frequently this behaviour is performed, the more likely that it becomes habitual^(^ ^26^ ^)^. When habits are formed, cognitive controlled behaviour transfers to automatic context-cued behaviour, reducing the demand on conscious processes^(^ ^27^ ^,^ ^28^ ^)^. Thus, when behaviour has a history of repetition in a stable context (e.g. eating popcorn in the cinema, eating a chocolate bar when feeling stressed), the context (cinema or feeling stressed), rather than a process of deliberation, may determine the behaviour^(^ ^29^ ^–^ ^31^ ^)^. Whereas deliberate decision making is mentally effortful, habits are cognitively efficient automatic behaviours which proceed without awareness and control^(^ ^26^ ^,^ ^27^ ^)^. Habit strength is a function of the frequency with which a specific behaviour has been repeated in a stable context and has acquired a certain degree of habitual automaticity^(^ ^32^ ^)^. Habit strength is considered an important predictor of several aspects of dietary behaviour in adults such as eating two or more fruits per day^(^ ^33^ ^)^, the number of sweets and chocolates consumed^(^ ^34^ ^)^, the frequency of binge alcohol consumption^(^ ^35^ ^)^, the number of unhealthy snack foods consumed^(^ ^36^ ^)^ and the energy intake from unhealthy snack foods^(^ ^23^ ^,^ ^37^ ^)^. However, the nature of the association between habit strength and snacking has not been explored for all types of between-meal snacks. As snacking behaviour varies across context and time, it is important to capture the fluctuating nature of momentary between-meal snacking in daily life. The experience sampling method (ESM), also known as ecological momentary assessment (EMA), is a structured self-assessment diary technique which allows to account for moment-to-moment within-person variability in snacking behaviour. Snackimpuls, a smartphone application (app) based on this method, was used to assess moment-to-moment between-meal snacking in real-life settings. A comparison study has demonstrated that the signal-contingent smartphone app was comparable with an estimated diet diary in assessing moment-to-moment energy intake from snacks^(^ ^38^ ^)^.

The studies mentioned above focus solely on specific food types or food groups. Focusing on strict food categories entails the risk of omitting important contributors to total energy intake from between-meal snacking. For instance, high-energy beverages which may not be experienced as satiating can nevertheless contribute to a substantial amount of surplus daily energy intake from between-meal snacks^(^ ^39^ ^–^ ^41^ ^)^. Moreover, snacks which are typically considered relatively innocent, such as artificially sweetened beverages and low-fat labelled snacks, can lead to overconsumption^(^ ^42^ ^,^ ^43^ ^)^ and consequently contribute to a substantial amount of energy intake from snacking. Furthermore, it is questionable whether individuals can adequately distinguish unhealthy from healthy snacks^(^ ^44^ ^,^ ^45^ ^)^. These findings seem to justify the inclusion of all types of between-meal snacks.

With regard to demographic differences, research has demonstrated that women^(^ ^46^ ^)^ are more likely to choose healthy snacks such as fruit, whereas men more often choose unhealthy snacks such as savouries. Men^(^ ^47^ ^)^ and young adults^(^ ^48^ ^)^ report more mean daily energy intake from snacks than women and older adults^(^ ^46^ ^)^, and a higher BMI has been associated with more unhealthy snack choices^(^ ^49^ ^)^. Finally, diet quality may differ by level of education. Adults with a high level of education tend to consume more fruits and vegetables compared with adults with other education levels^(^ ^50^ ^–^ ^52^ ^)^ and show a higher variability in nutrient content, which is an indicator for a better diet quality^(^ ^52^ ^)^. As such, research investigating the association between habit strength and different categories of snacks often controls for respondents’ gender, age, level of education and BMI^(^ ^33^ ^,^ ^35^ ^,^ ^37^ ^)^. Verhoeven *et al.* ^(^ ^23^ ^)^ examined the moderating role of these respondent characteristics on the association between habit and mean daily energy intake from unhealthy snack foods in a community sample. No interaction effects were found. It is unknown, however, whether or not these demographic characteristics act as moderators on the association between habit and momentary energy intake from all types of between-meal snacks. Identification of such moderators is critical to develop effective tailored health intervention programmes targeting habitual snacking.

To summarize, the present study investigates the association between habit strength and moment-to-moment energy intake from all types of between-meal snacks. It is hypothesized that habit strength is significantly associated with moment-to-moment energy intake from between-meal snacks in daily life. In addition, the study examines whether gender, age, level of education and BMI moderate the association between habit strength and momentary energy intake from snacks.

## Materials and methods

### Sample

Participants were recruited throughout the Netherlands via social media, websites and newsletters, and within the networks of several master thesis students at the Open University of the Netherlands. In total, 468 adults of the general population agreed to take part in the present study.

**Fig. 1:**
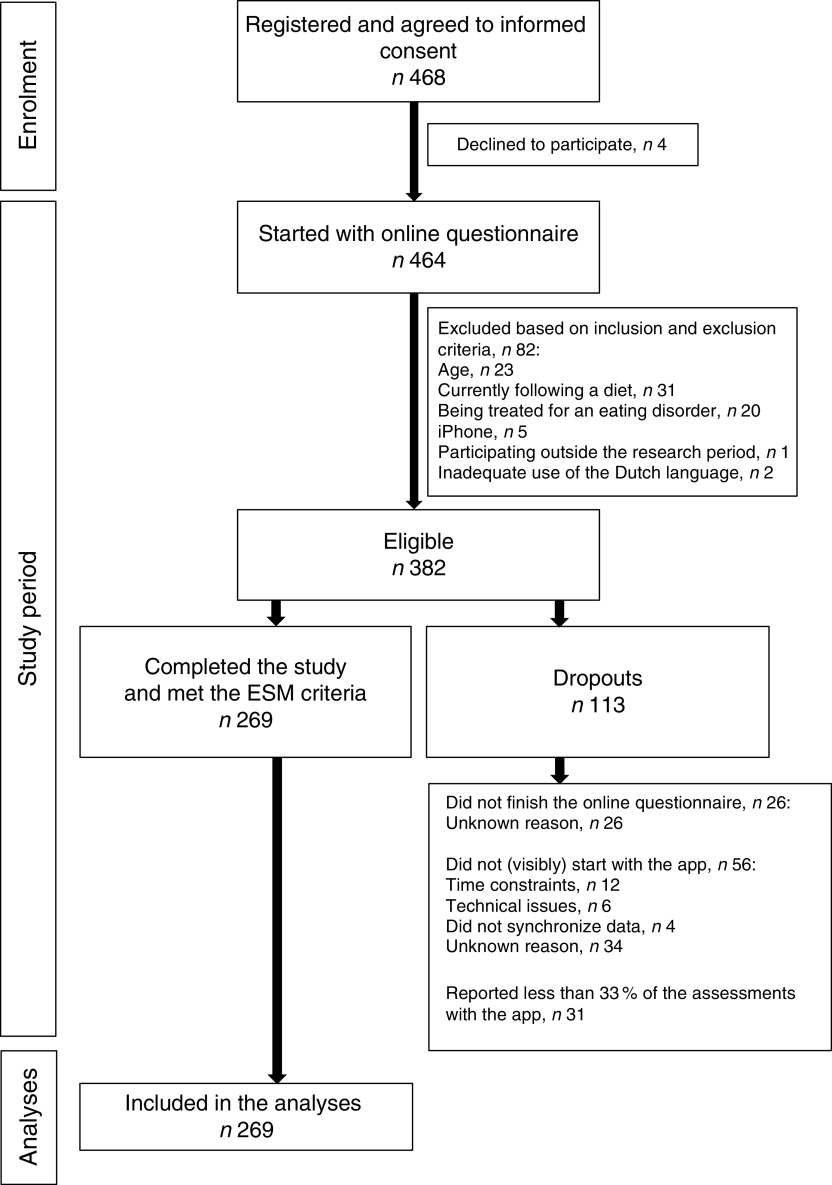
Study flowchart (ESM, experience sampling method)

Individuals had to be 20–50 years of age to be included in the analyses, as research has shown the largest increase in overweight individuals in recent years within this age group in the Netherlands^(^ ^53^ ^,^ ^54^ ^)^. Participants had to be in possession of an Android smartphone as the Snackimpuls app was available only for this platform. Exclusion criteria were currently following a diet, being treated for an eating disorder in the present or the past, participating outside the research period (see ‘Procedure’ below) and unfamiliarity with the Dutch language. There were no criteria regarding BMI. Based on these criteria, 382 of the 468 participants were eligible to participate in the present study (Fig. 1).

By registering for the research all participants agreed to an informed consent and authorized the researcher to use the data of the research for scientific purposes. The participants also agreed to accept the terms and conditions as formulated in a licence statement. It was emphasized that participation in the research was entirely voluntary and that participants had the right to discontinue the research at any time. Participants were informed that all data would be processed anonymously and that the answers would be handled confidentially. The Ethics Committee of the Open University of the Netherlands approved the research (reference number U2014/03151/HVM).

### Procedure

The research took place in the Netherlands in the period from mid-October 2012 until early December 2013. Respondents were enrolled for one week during the research period, were instructed to take part during a regular week excluding holidays and were to maintain their usual food intake.

The Snackimpuls app, and the Snackimpuls website which contains information and instructions for participants, were created for the present study by the Open University of the Netherlands. Recruited participants were referred to the website to obtain more information about the study, including instructions for downloading and installing the Snackimpuls smartphone app. After registration at the website, participants automatically received an email with a link to an online questionnaire. Having completed this questionnaire, participants automatically received login credentials for the free smartphone app. A demo version was included in the smartphone app as a training opportunity on the day prior to the start of the assessment period. Each day during the seven-day research period, respondents repeatedly answered a short questionnaire (thirty-seven items) on their smartphone to collect multiple assessments (ten times per day) of current emotions, self-esteem, situational and social context, and between-meal snack intake. This questionnaire took approximately 5 min to complete. In addition, each day participants answered a brief self-initiated questionnaire on their smartphone after waking up (four items) and before going to bed (ten items). After waking up, respondents’ quality of sleep was assessed. Before going to bed, questions were asked about respondents’ reflective assessments of the past day. In addition, between-meal snack intake was assessed one last time, to cover late-night snacking.

Finally, participants were instructed to synchronize the data on their smartphone with the main server of the Snackimpuls project at the end of their research period. To enhance compliance, participants were able to contact a member of the research team by email in case of questions or problems.

Three Android tablets were raffled off among the participants as a reward. All participants received personal feedback based on their individual scores regarding eating behaviour^(^ ^55^ ^)^, daily activities and affective states (Snackimpuls app).

### Instruments

Two instruments were used to collect the data. First, at baseline, an online composite questionnaire was used to collect data on demographics and habit strength. Subsequently the smartphone app Snackimpuls was used to collect repetitive data of between-meal snack intake. Since the present study was part of a larger study to investigate determinants of between-meal snacking in daily life^(^ ^38^ ^,^ ^56^ ^–^ ^58^ ^)^, other concepts (not used in the current study) were assessed as well with the online questionnaire (e.g. personality) and with the Snackimpuls app (e.g. ego depletion, quality of sleep).

#### Online composite questionnaire

Demographic variables, such as age, weight, height, gender, marital status and the highest completed level of education, were assessed. In the present study, level of education was categorized as high education (higher vocational or academic education) *v.* low to middle education (low: none, elementary school or lower general education; middle: intermediate general education, intermediate vocational education, higher general secondary education or pre-university education). BMI was calculated as weight (in kilograms) divided by the square of height (in metres).

Habit strength was assessed with the valid and reliable Self-Report Habit Index (SRHI)^(^ ^26^ ^)^. The SRHI is currently the most commonly used measure of habit strength in health behaviours^(^ ^21^ ^,^ ^59^ ^)^. The SRHI consists of twelve responses to a generic stem: ‘Doing behaviour X is something …’ (e.g. ‘I do automatically’, ‘I do without thinking’, ‘I start doing before I realize I’m doing it’). For the present study’s purpose, the stem of the SRHI was adapted to refer to the habit of eating and drinking between meals (e.g. ‘Eating and drinking between meals is something …’). Each item was scored on a 5-point Likert scale from 1 (‘totally agree’) to 5 (‘totally disagree’). Afterwards, all items were recoded to facilitate interpretation: a higher score reflects a higher habit strength of between-meal snacking. In the current study, the SRHI showed excellent internal consistency (Cronbach’s *α*=0·92).

#### The experience sampling smartphone app

Between-meal snack intake was assessed in daily life with the ESM^(^ ^60^ ^,^ ^61^ ^)^, a structured self-assessment diary method. The Snackimpuls app produced ten audio quasi-random signals (beeps) per day for seven consecutive days between 07.30 and 22.30 hours, prompting participants to report. The beeps had an average interval of 90 min (range 21–159 min) and were programmed at a random moment in each of the ten 90-min time blocks per day. Respondents were instructed to complete the reports immediately after the signal.

In the current study, between-meal snacks were defined as all types of consumptions (e.g. chocolate, grapes, orange juice) other than main meals (i.e. breakfast, lunch and dinner). Since assessments are conducted at quasi-random times with an average interval of 90 min, the reported between-meal snack intake with the Snackimpuls app encompasses an average time frame of 90 min (snack intake since the former beep). Regarding between-meal snacking, participants answered the question: ‘Did you eat or drink anything between meals since the last beep?’ by replying ‘Yes’ or ‘No’. If the reply was negative this was equated with 0 kcal. If the answer was affirmative, they were asked to report every product consumed and its quantity. To help participants facilitate the recording of snack intake, the Snackimpuls app has a built-in search function. This search function consults a food composition table based on the scientifically accepted Dutch Food Composition Database^(^ ^62^ ^)^. For every reported snack, participants chose between two quantity options. Natural products, such as an apple, and products with standardized quantities, such as a Mars candy bar, could be reported either per piece or in grams (for solid foods) or millilitres (for fluids). Products with undetermined quantities such as yoghurt or tea could be reported in relevant household measurements (i.e. a bowl or a cup) or in grams or millilitres. The snack intake was automatically converted into kilocalories. This information was not visible to the participants. Products that were not available in the search facility could be easily added by the participants using the keyboard of their smartphone. These self-added reported snacks were converted into their corresponding kilocalories by two independent researchers. The kilocalories for these products were extracted from the scientifically accepted Dutch Food Composition Database^(^ ^62^ ^)^. If reported products were not available in the Dutch Food Composition Database, the database of the Netherlands Nutrition Centre^(^ ^63^ ^)^ was consulted. In addition to assessments prompted by the audio signals, between-meal snack intake was also assessed by a daily self-initiated short questionnaire just before going to bed.

A pilot study has demonstrated the feasibility and usability of the Snackimpuls app^(^ ^56^ ^)^.

### Statistical analyses

Because ESM data have a hierarchical structure with repeated momentary measurements (level 1) for each participant (level 2), multilevel linear techniques were used.

Statistical analyses were performed to evaluate which model best fit the data (i.e. fixed or random slopes). Subsequently, multilevel linear regression analyses were carried out using the xtmixed procedure in Stata/MP version 11 (2009). The key variables were standardized prior to the analyses. After standardization, the associations could be directly assessed and their importance was evaluated by using the calculated regression coefficients (*β*). A multilevel regression analysis was conducted to examine the association between habit strength and energy intake from between-meal snacks in daily life. The analysis was adjusted for potential confounders. To determine whether the variables gender, age, level of education and BMI moderate the association between habit strength and energy intake from snacks, standardized interaction variables were created and included in the analyses. The level of significance for all analyses was defined at *P*<0·05. To determine inter-rater reliability for the assigned kilocalories to the reported snack consumptions which were not available in the search facility, bivariate correlations (Pearson’s *r*) between the ratings were calculated.

Missingness in the current study occurred at beep level, which is a known phenomenon in ESM research^(^ ^64^ ^)^. Participants were instructed to complete their reports immediately after the beep, to minimize memory distortion. Reports not completed within 15 min after the beep were considered invalid. Participants were considered valid if they had reported at least 33 % of the total number of assessments with the app during the seven-day research period, within 15 min after the beep. This is in accordance with previous ESM research which showed that a minimum of 33 % response to the predefined protocol was required to obtain valid data^(^ ^65^ ^)^. Since snack intake could be reported eleven times per day (ten momentary reports and the final report just before going to bed to cover late-night snacking) for seven consecutive days, participants with fewer than twenty-six valid reports were considered dropouts. Dropout analyses were conducted (two-sample Wilcoxon rank-sum (Mann–Whitney) tests) to investigate significant differences in habit strength, age and BMI between participants who finished the study and the dropouts. Effect sizes were expressed as correlation coefficients (Pearson’s *r*)^(^ ^66^ ^)^. To investigate significant differences in the distribution of gender and level of education between these two groups, *χ* ^2^ analyses were conducted.

## Results

Of the total sample that participated in the study (*n* 464), eighty-two respondents (18 %) did not meet the inclusion criteria. Of the eligible sample (*n* 382), 113 participants (30 %) dropped out and 269 participants (70 %) completed the study (Fig. 1). Dropouts (*n* 113) did not differ from the participants who finished the study (*n* 269) with respect to BMI (*Z*=−0·26, *P*=0·80). Moreover, no significant differences were found in the distribution of gender (*χ* ^2^ (1, *n* 382)=0·91, *P*=0·34) and level of education (*χ* ^2^ (1, *n* 382)=0·01, *P*=0·94) between both groups. However, dropouts were slightly younger (mean age: 33 *v.* 35 years; *Z*=2·67, *P*=0·01). The effect size of this finding was small (*r*=0·14). Dropouts also had a slightly higher habit strength (mean habit: 3·11 *v.* 2·83; *Z*=−2·65, *P*=0·01). The effect size of this finding was small (*r*=0·14). Data from the completers were included in the analyses, except data from respondents (*n* 2) who consistently did not report any type of snack consumed. Mean age of the completers (197 females, 73 %; seventy males, 27 %) was 35 (sd 8·91) years (range: 20–50 years) and mean BMI was 24 (sd 4·00) kg/m^2^ (range: 17–43 kg/m^2^). Of the participants, 61 % had a higher vocational or academic degree (Table 1).

**Table 1:** Sociodemographic and habit strength characteristics of the study participants: adults aged 20–50 years (*n* 269), the Netherlands, October 2012 to December 2013

Characteristic	*n*	%	Mean	sd	Range	
Gender	269					
Male	72	27				
Female	197	73				
Age (years)	269		35·42	8·91	20–50	
BMI (kg/m^2^)	267		24·39	4·00	17–43	
<18·5	4	1				
185≤BMI<25·0	162	61				
25·0≤BMI<30·0	79	30				
≥30·0	22	8				
Education*	269					
High	163	61				
Low to middle†	106	39				
Variable	*n*	%	Mean	sd	Range	Cronbach’s *α*
SRHI	269		2·83	0·81	1–5	0·92

SRHI, Self-Report Habit Index.

* High education: higher vocational or academic education. Low to middle education: none, elementary school, lower general education, intermediate general and intermediate vocational education, higher general secondary or pre-university education.

† Combined groups because of the small sample size in the low level of education group (*n* 11).

Snack intake could be reported eleven times per day (ten momentary reports and the final report just before going to bed to cover late-night snacking). Study participants yielded 14 330 momentary reports, 69 % of the maximum number of assessments (11 reports×7 days×269 participants) with the Snackimpuls app. In 7174 assessments (50 %) participants indicated that they did consume between-meal snacks. However, snack intake was missing at 572 assessments: although respondents indicated they did consume something between-meals, no products were reported (Table 2).

**Table 2:** Momentary reports (*n* 14 330) logged by the study participants: adults aged 20–50 years (*n* 269), the Netherlands, October 2012 to December 2013

	*n*
Snack consumption: no	7156
Snack consumption: yes	7174
With energy (kilocalories)	5198
Without energy (kilocalories; e.g. water, black coffee)	1404
No products reported	572

The 6602 momentary reports of between-meal snack intake (with and without kilocalories) comprised 11 520 reported between-meal snacks of which 9593 snacks (83 %) were reported with the search facility of the app and 1927 snacks (17 %) were reported manually. Inter-rater reliability yielded high correlation coefficients (*r*=0·95, *P*<0·01). In 7156 assessments (50 %) participants indicated that they did not consume any between-meal snacks. If snacks were reported, between-meal snacking resulted in a mean momentary energy intake per respondent of 162 (sd 216) kcal (678 (sd 904) kJ).

Results from the completers showed a significant main effect of habit strength on moment-to-moment between-meal snack intake (*β*=0·05 (se 0·02), *P*<0·01): the higher the strength of habit to snack between meals, the higher the amount of energy consumed. In addition, results of the interaction analyses revealed no significant interaction between habit strength and gender (*β*=0·09 (se 0·09), *P*=0·31), habit strength and age (*β*=0·03 (se 0·09), *P*=0·75) or habit strength and BMI (*β*=0·16 (se 0·13), *P*=0·21). However, a significant interaction was found between habit strength and level of education (*β*=−0·23 (se 0·08), *P*<0·01) in association with momentary energy intake from snacks. Additional multilevel regression analyses stratified by level of education showed that the association between habit strength and momentary energy intake from between-meal snacks was significant (*β*=0·11 (se 0·03), *P*<0·01) in the low to middle level of education group.* In the high level of education group, there was no significant association between habit strength and energy intake from between-meal snacks (*β*=0·00 (se 0·02), *P*=0·99).

## Discussion

The aim of the present study was to investigate the association between habit strength and energy intake (kilocalories) from between-meal snacking in daily life. The study also examined whether gender, age, level of education and BMI moderated the association between habit strength and momentary energy intake from snacks.

Results showed that habit strength was significantly associated with momentary energy intake from between-meal snacks in daily life: the higher the strength of habit to snack between meals, the higher the amount of energy consumed at beep level. In addition to previous studies focusing solely on strict food categories, the present study demonstrates that habit strength predicts energy intake from between-meal snacks in the broader sense (i.e. all types of consumptions other than main meals). The current study contributes to existing findings which have demonstrated the role of habit strength in predicting different aspects of nutrition (e.g. alcohol consumption, fruit consumption, snack food consumption, energy intake from unhealthy snacks).

Results showed no moderating role of gender, age and BMI on the association between habit strength and energy intake from snacks. In the present study, habit strength exerted the same influence on energy intake from between-meal snacking in men and women, in different ages within the scrutinized age group (20–50 years) and in individuals with different BMI. This seems to indicate that there is no need to differentiate between these demographic subgroups in interventions targeting habitual snacking. However, research has demonstrated that contextual cues (i.e. being in the presence of others, being alone) that may trigger habitual snack intake differ according to BMI^(^ ^67^ ^)^. In such cases, interventions may still need to differentiate between demographic subgroups.

In the present study, the association between habit strength and momentary energy intake from snacks was moderated by level of education. Additional analyses showed that habit strength was significantly associated with moment-to-moment energy intake from between-meal snacks in daily life in the low to middle level of education group; there was no significant association between habit strength and momentary energy intake from between-meal snacks in the high level of education group. This finding is in contrast with the study of Verhoeven *et al.* ^(^ ^23^ ^)^ which showed no moderating role of level of education. The discrepancies between the findings of both studies may be due to differences in the definition of snacking (between-meal snacks *v.* unhealthy snack foods), the sampling procedure of snack intake (repeated sampling during the day *v.* once per day at the end of the day) and the measurement level (moment-to-moment energy intake *v.* mean daily energy intake).

Previous research has pointed out that diet quality may differ by level of education. Adults with a high level of education tend to consume more fruits and vegetables compared with adults with other education levels^(^ ^51^ ^,^ ^52^ ^)^ and show a higher variability in nutrient content, which is an indicator for a better diet quality^(^ ^52^ ^)^. A low to middle level of education is associated with more unhealthy dietary behaviour^(^ ^68^ ^,^ ^69^ ^)^, less nutritional knowledge^(^ ^45^ ^,^ ^51^ ^)^, a higher concern with costs and a lower concern with health aspects in food choices^(^ ^69^ ^,^ ^70^ ^)^. How does this relate to our findings? It is conceivable that low- to middle-educated individuals make different cognitive assessments regarding their snacking behaviour compared with high-educated individuals. It may be that low- to middle-educated individuals are less proficient in making cognitive assessments in accordance with health aspects. As cognitive assessments precede habit formation, it is plausible that disparities in cognitive assessments between the level of education groups lead to differences in habitual snacking. Indeed, when habits are formed, cognitive controlled behaviour transfers to context-cued automatic behaviour^(^ ^28^ ^,^ ^71^ ^)^. Furthermore, it is suggested that individuals with a low to middle level of education are more vulnerable to the temptation of the immediate rewards that might accompany highly palatable and energy-dense snacks^(^ ^72^ ^)^. This vulnerability may eventually lead to the development of high-energy snacking habits, as it has been shown that rewarding experiences are one of the features facilitating the formation of habits^(^ ^24^ ^–^ ^26^ ^,^ ^73^ ^)^.

Some limitations of the present study have to be noted. First, the sample of the current study was not representative for the general population because it was biased in favour of women and individuals with a high level of education. However, our results lead us to the conclusion that we are still able to provide relevant findings for both the high- and low- to middle-educated individuals. The small number of participants with a low level of education adds to the limitations of the current study. However, our results show that both in the low and the middle education group there is a significant association between habit and energy intake from snacks (see footnote). This association is even stronger for individuals with a low level of education. As a consequence, our finding on the combined level of education group may be considered conservative due to the small number of low-educated individuals. Second, the dropouts in the study were slightly younger and had a slightly higher habit strength than completers. Third, in the present study, between-meal snacking was assessed using self-reports, which are vulnerable to incomplete data and/or under-reporting^(^ ^74^ ^,^ ^75^ ^)^. Nevertheless, the compliance rate of the current study is consistent with compliance rates in previous ESM studies in similar samples^(^ ^64^ ^)^. In addition, we conducted a comparison study (*n* 46)^(^ ^38^ ^)^ which showed that momentary energy intake reported with the Snackimpuls app was comparable to the reports with an estimated diet diary, which is considered effective in assessing dietary intake^(^ ^76^ ^,^ ^77^ ^)^. Fourth, in our study the distinction between snacking and main meals (breakfast, lunch and dinner) relies on participants’ individual classification of whether a consumption was a snack or part of a meal. This can be considered a limitation of the study. Indeed, it has been pointed out that respondents may use different criteria for classification such as the time of day or the type of consumption^(^ ^13^ ^)^. Fifth, although the use of the SRHI in our study can be considered a strength, it may be that habitual behaviour fluctuates over time and across situations. Based on our findings, future research may consider investigating habitual snacking behaviour related to specific, predefined contexts in low- to middle-educated adults, since actual habitual snacking behaviour may fluctuate over time and across situations. Sixth, our study does not account for differences in habit strength between certain foods and beverages. Sensory research has identified individual differences in taste preferences which may lead to preferential snack choices based on sensory features^(^ ^78^ ^)^. It has been demonstrated that individuals who prefer a sour taste consume fruit more often, whereas sweet snacks such as chocolate are more often consumed by individuals who prefer a sweet taste^(^ ^79^ ^)^. As such, when habits are developed these individual differences may lead to a higher dose of consumption of particular snacks^(^ ^80^ ^)^ or snacks with a particular taste. However, since in daily life all types of consumptions may be consumed, our results lead us to the conclusion that we were still able to provide relevant findings on the impact of habit on energy intake from snacks. Nevertheless, the differential effect of habitually consuming specific types of consumptions remains an important endeavour for future research. For example, intervention studies targeting unhealthy habitual snacking may consider increasing the liking of sour tastes using preference conditioning strategies^(^ ^81^ ^)^. Finally, the outcome measure in the current study was energy intake (kilocalories), which contributes to overweight and obesity. However, since main meals were not included, our results may not reflect total habitual energy intake. Still, our findings do shed light on one of the major sources of weight gain and obesity^(^ ^8^ ^–^ ^12^ ^)^. Additional analyses to verify if beeps in which only healthy products were reported (i.e. fruit and/or vegetables) might have influenced the results showed that the findings were similar when these beeps (*n* 245) were excluded from the analyses. Moreover, additional analyses to verify if beeps in which only beverages were reported might have influenced the results showed that the findings were similar when these beeps (*n* 2738) were excluded from the analyses.

Despite these limitations, several strengths of the present study also have to be mentioned. First, to our knowledge, the current study was the first to investigate the association between habit strength and energy intake from snacking in daily life including all types of between-meal snacks. Second, our sample represents the age group in which the largest increase in overweight has been shown in recent years in the Netherlands^(^ ^53^ ^,^ ^54^ ^)^. The largest health gain may be achieved in this segment of the population.

The findings of the present study may have implications for the development of interventions. According to our results, interventions should aim at reducing energy intake from habitual snacking in low- to middle-educated individuals. It has been pointed out that traditional behaviour change interventions are less successful in modifying habitual behaviours, as habits are resistant to change^(^ ^22^ ^)^. In addition to the three general principles of behaviour change (i.e. making a decision to take action, translating the decision into action, perpetuating the new behaviour), habit formation requires a fourth principle: the new action must be repeated in stable contexts^(^ ^24^ ^)^. Research has identified effective intervention strategies to alter dietary habits, such as the use of reminders, self-monitoring and self-control, cue awareness, implementation intentions and mental contrasting^(^ ^24^ ^,^ ^82^ ^,^ ^83^ ^)^. However, research towards the effectiveness of these strategies in individuals with low to middle levels of education is still rather limited^(^ ^22^ ^,^ ^27^ ^,^ ^84^ ^)^ and could be further reinforced.

When designing interventions targeting dietary habits in low- to middle-educated individuals, there are several issues to consider. Altering dietary habits requires linking a critical contextual cue for an unhealthy snack response to a healthier alternative. Repeatedly replacing the old behaviour (e.g. eating a bar of chocolate) by the new behaviour (e.g. eating an apple) in a stable context is one of the requisite pathways to alter dietary habits. As individuals with a low to middle level of education have less nutritional knowledge^(^ ^45^ ^,^ ^51^ ^)^, it is recommended to include examples of alternative healthy snacks in the intervention to avoid replacing an unhealthy snack by another unhealthy snack (e.g. replacing a chocolate bar as the habitual snack by peanuts as the alternative, or replacing soft drinks by fruit juices)^(^ ^83^ ^,^ ^85^ ^)^. Considering the higher concern with costs of individuals with a low to middle level of education^(^ ^69^ ^,^ ^70^ ^)^, emphasizing the relatively low costs of certain healthy alternatives may help to overcome this barrier. Research has also shown that rewarding experiences are one of the features facilitating habit formation^(^ ^24^ ^–^ ^26^ ^,^ ^73^ ^)^. As individuals with a low to middle level of education are more vulnerable to the temptation of immediate rewards^(^ ^72^ ^)^, enhancing self-control and stimulating self-monitoring may aid altering snacking habits. Another suggested pathway is reward conditioning through reinforcement. Mere exposure to a less appreciated healthy snack, coupled with a rewarding incentive, may increase liking and thereby contribute to healthy habit formation^(^ ^73^ ^,^ ^86^ ^,^ ^87^ ^)^.

In addition to specific habit features, general aspects of dietary behaviour change should also be addressed. Research has already demonstrated that low- to middle-educated individuals are less proficient in converting information into appropriate health behaviour^(^ ^88^ ^,^ ^89^ ^)^. Interventions in which complex nutritional information is limited are recommended^(^ ^90^ ^)^. It has also been suggested that participants with low to middle levels of education may need external support (i.e. guidance through dietary interventions), whereas high-educated individuals may make dietary improvements on their own, based on publicly available information^(^ ^91^ ^)^. Low- to middle-educated individuals may need their friends, families and peers in order to create social support to alter their dietary habits^(^ ^92^ ^,^ ^93^ ^)^. Interventions incorporating features such as personalized feedback, reminders and/or opportunities for personal contact should be considered^(^ ^94^ ^)^.

## Conclusion

The present study contributes to our understanding of habitual energy intake from snacks. Results show that habit strength is an important predictor of energy intake from between-meal snacks in an adult population sample. The impact of this association was found to be similar in terms of gender, age and BMI, but not for level of education. The influence that habit strength exerts on between-meal snack intake is significant for low- to middle-educated individuals, and non-significant for individuals who attained a high level of education. Based on the findings of the current study it is recommended to address habitual between-meal snacking in future interventions targeting low- to middle-educated individuals.

## Footnotes

* Low level of education (*n* 11): *β*=0·14 (se 0·05), *P*<0·01; middle level of education (*n* 96): *β*=0·10 (se 0·03), *P*<0·01.
